# Perfusion of surgical cavity wall enhancement in early post-treatment MR imaging may stratify the time-to-progression in glioblastoma

**DOI:** 10.1371/journal.pone.0181933

**Published:** 2017-07-21

**Authors:** Ji Eun Park, Kyoung Hwa Ryu, Ho Sung Kim, Hyo Won Kim, Woo Hyun Shim, Seung Chai Jung, Choong Gon Choi, Sang Joon Kim, Jeong Hoon Kim

**Affiliations:** 1 Department of Radiology and Research Institute of Radiology, University of Ulsan College of Medicine, Asan Medical Center, Seoul, Korea; 2 Department of Radiology, Gyeongsang National University School of Medicine, Gyeongsang National University Changwon Hospital, Changwon, Republic of Korea; 3 Department of Neurosurgery, University of Ulsan College of Medicine, Asan Medical Center, Seoul, Korea; Universita degli Studi di Palermo, ITALY

## Abstract

**Objective:**

To determine if perfusion in surgical cavity wall enhancement (SCWE) obtained in early post-treatment MR imaging can stratify time-to-progression (TTP) in glioblastoma.

**Materials and methods:**

This study enrolled 60 glioblastoma patients with more than 5-mm-thick SCWEs as detected on contrast-enhanced MR imaging after concurrent chemoradiation therapy. Two independent readers categorized the shape and perfusion state of SCWEs as nodular or non-nodular and as having positive or negative perfusion compared with the contralateral grey matter on arterial spin labeling (ASL). The perfusion fraction on ASL within the contrast-enhancing lesion was calculated. The independent predictability of TTP was analyzed using the Kaplan-Meier method and Cox proportional hazards modelling.

**Results:**

The perfusion fraction was higher in the non-progression group, significantly for reader 2 (*P* = 0.03) and borderline significantly for reader 1 (*P* = 0.08). A positive perfusion state and (*P* = 0.02) a higher perfusion fraction of the SCWE were found to become an independent predictor of longer TTP (*P* = 0.001 for reader 1 and *P* < 0.001 for reader 2). The contrast enhancement pattern did not become a TTP predictor.

**Conclusion:**

Assessment of perfusion in early post-treatment MR imaging can stratify TTP in patients with glioblastoma for adjuvant temozolomide therapy. Positive perfusion in SCWEs can become a predictor of a longer TTP.

## Introduction

Surgical cavity wall enhancement (SCWE) indicates a nonmeasurable lesion according to the Response Assessment in Neuro-Oncology (RANO) Working Group [1], unless any measurable criteria exist. Previous assessments of nonmeasurable and measurable SCWEs using early postoperative MR imaging to evaluate remnant tumor and prognosis in patients with glioblastoma [2–4] revealed that thick, nodular SCWEs are associated with a poorer prognosis compared with lesions showing thin or linear enhancement [2, 4]. However, because of the narrow time window of MR imaging, no clinical study to date has reported on the significance of SCWEs on MR imaging obtained after concurrent chemoradiation therapy (CCRT) but before adjuvant temozolomide (TMZ) therapy (early post-CCRT MR imaging).

From early post-CCRT MR imaging analysis, the microenvironment of SCWEs has been shown to be complex, containing a mixture of radiation necrosis, recurrent tumor, parenchymal gliosis, and ‘inactive’ neoplasm [5]. Arterial spin labeling (ASL) appears to be a promising tool for perfusion evaluation [6–8], offering a strong radiology–pathology correlation [9] and the advantage of being a completely noninvasive method that uses an endogenous tracer from inflowing arterial blood. ASL imaging has been used to differentiate pseudoprogression and recurrent tumor in the early post-CCRT state [10, 11], with pseudoprogression or radiation necrosis showing decreased perfusion and recurrent tumor showing increased perfusion.

Although increased perfusion is generally associated with increased tumor vascularity [6, 9, 12], recent translational research has proposed that increased perfusion may be an indicator of normalized tumor vessels that can alleviate hypoxia and improve drug delivery to tumors [13–16]. On the other hand, immature tumor vessels can lead to a heterogeneous pattern of tumor perfusion [17], ineffective tumor blood supply, and reduced effective drug delivery [15]. We hypothesized that the perfusion status of SCWEs in the early post-CCRT state determined using ASL may be predictive of the time-to-progression (TTP) because low perfusion would reduce the effectiveness of subsequent adjuvant chemotherapy treatment. In addition, the enhancement pattern of SCWEs found on early post-CCRT MR imaging would help to evaluate the clinical significance of SCWEs. Thus, the purpose of our study was to determine if perfusion in surgical cavity wall enhancement (SCWE) obtained with early post-treatment MR imaging could be used to stratify the time-to-progression (TTP) in glioblastoma.

## Methods

### Patient selection

**Fig 1:**
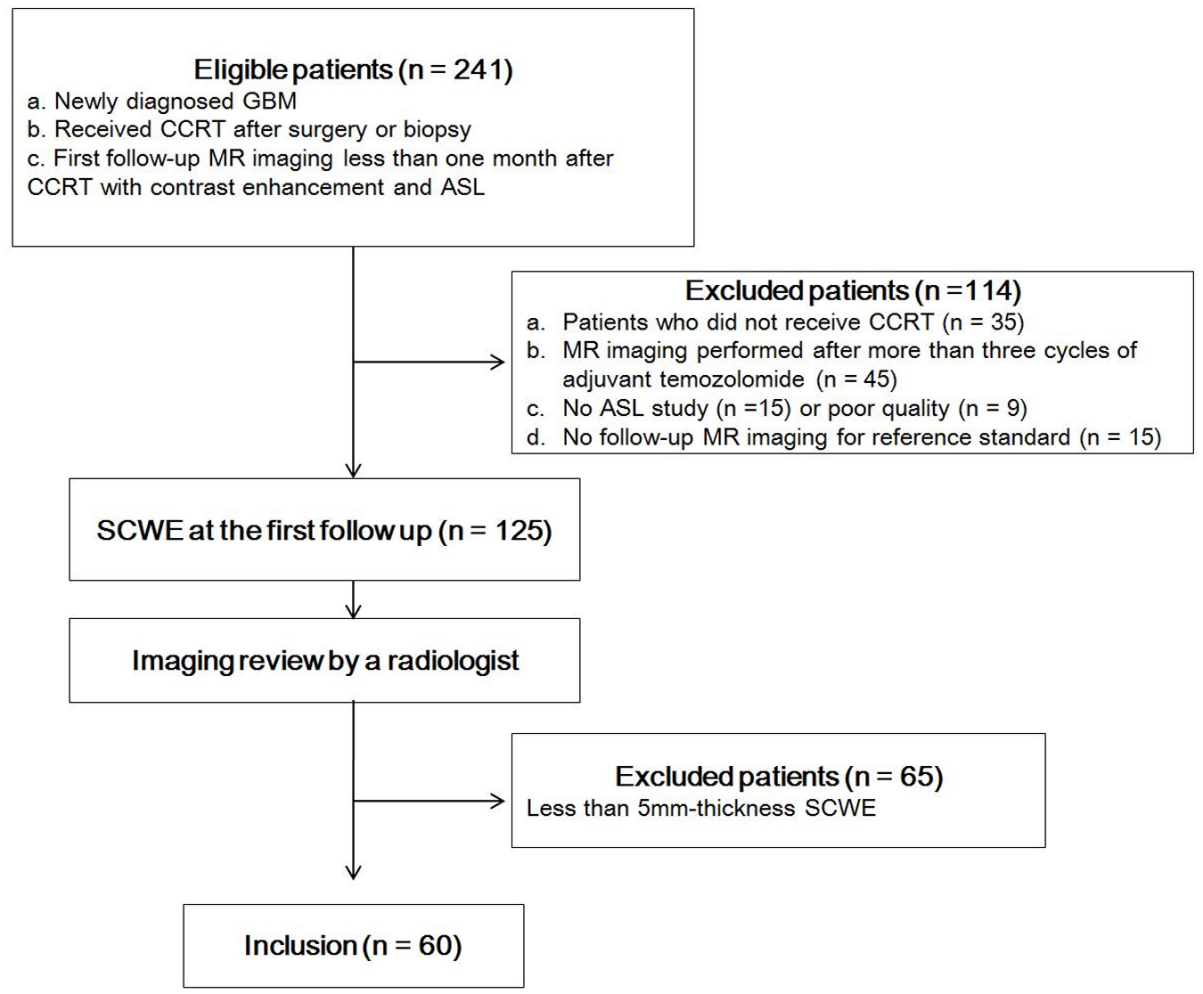
Flow diagram of patient selection. SCWE = surgical cavity wall enhancement.

Our institutional review board approved this retrospective study and waived the need for informed consent. Two hundred and forty-one consecutive patients with newly diagnosed glioblastoma who had undergone surgical resection or stereotactic biopsy from August 2010 to July 2016 at our institution were identified from our neuro-oncologic database. The inclusion criteria were as follows: (a) histopathologically proven newly diagnosed glioblastoma; (b) 12-week treatment with the standard CCRT ([60 Gy administered as 2-Gy fractions 5 days per week] and oral temozolomide [TMZ; 75 mg per square meter of body surface area per day for a maximum of 49 days]) regimen; and (c) MR imaging with contrast enhancement and ASL within 1 month of CCRT completion. The exclusion criteria were: (a) no CCRT or adjuvant TMZ (n = 35); (b) MR imaging performed at more than 1 month after CCRT (n = 45); (c) no ASL sequence performed (n = 15); (d) no follow-up MR imaging for use as a reference standard (n = 15); and (e) poor quality ASL imaging (n = 9). Among them, patients with thin SCWE less than a 5-mm-thick were further excluded (n = 67) because an assessment of ASL in these lesions is not appropriate. A final total of 60 patients was included in the study. The patient accrual process is summarized in Fig 1.

### Reference standards

Tumor progression was determined by our neuro-oncology team (consisting of radiologists and neurosurgeons) according to RANO criteria [1, 18]. The neuro-oncology team recorded the clinical characteristics of the patients, including age, sex, Karnofsky performance score (at the first follow-up MR imaging session after CCRT), surgical extent (biopsy, partial resection, or gross total resection), number and dose of adjuvant TMZ cycles, time between the first follow-up imaging and start of the adjuvant TMZ, and the TTP from the date of the first follow-up imaging.

### Image acquisition

All MR imaging was performed on a 3-T MR scanner (Achieva; Philips Medical Systems, Best, The Netherlands) using an eight-channel head coil. The protocol included T2-weighted imaging, fluid-attenuated inversion recovery imaging, diffusion-weighted imaging, ASL, DSC perfusion MR imaging, and precontrast T1-weighted imaging (T1WI). Contrast enhancement was achieved with 0.1 mmol/kg gadoterate meglumine (Dotarem; Guerbet, Paris, France) after a fat-suppression pulse.

Acquisition of two-dimensional pseudo-continuous ASL was performed using multi-shot spin-echo echo-planar imaging. Imaging parameters were as follows: labeling time, 1800 ms; labeling width, 130 mm; repetition time, 3000 ms; echo time, 13 ms; no vascular crushing; acquisition matrix, 64 × 59; acquisition voxel size, 3.44 × 3.67 mm; reconstruction matrix, 128 × 128; reconstruction voxel size, 1.72 × 1.72 × 6 mm; field of view, 220 × 220 × 104 mm; 15 slices of 6-mm thickness with a 1-mm slice gap; SENSE factor, 2.3; whole brain coverage; and a total scan time of 5 min. After motion correction of the ASL images, ASL imaging data were digitally transferred from the PACS workstation to a personal computer.

### Qualitative analysis of SCWEs

SCWEs on post-CCRT MR imaging were evaluated by two radiologists (H.S.K. and J.E.P. with 17 years and 5 years of experience in neuro-oncology MRI, respectively) who were blind to the patient histories. The readers were asked to independently evaluate each SCWE for the following: (1) the contrast enhancement pattern (nodular or non-nodular); (2) the presence of measurable contrast-enhancing lesions at two maximal perpendicular diameters of the SCWE in the axial plane; and (3) positive or negative perfusion at the SCWE.

The enhancement pattern of the SCWE was categorized as either non-nodular, when the wall enhancement was thickened but without definite nodular enhancement, or nodular, when there was a nodular enhancement of ≥ 5 mm at two maximal perpendicular diameters. The presence of measurable contrast-enhancing lesion was recorded when the SCWE included a bidimensional contrast-enhancing lesion with two perpendicular diameters of at least 10 mm [1].

The perfusion state was assessed in patients with a ≥ 5-mm-thick contrast-enhanced SCWE because the acquisition voxel size of ASL was 3.44 × 3.67 mm and the minimum size for perfusion evaluation was at least 4.00 mm. For perfusion assessment, the two readers visually assessed using ASL whether there was positive or negative perfusion in the SCWE compared with normal-appearing contralateral cortical grey matter. Positive perfusion was defined when as the tumor showing a similar to increased perfusion compared with normal-appearing cortical grey matter [6, 12, 19].

Each reader recorded the SCWE category but the final enhancement pattern was determined by consensus to resolve disagreements and to improve reproducibility, as suggested in a previous study [4]. Before consensus, the kappa value for each category was as follows: (1) enhancement pattern 0.84 (95% confidence interval [CI] = 0.70–0.99), (2) presence of measurable contrast-enhancing lesions 1.00, and (3) determination of positive or negative perfusion 0.84 (95% CI = 0.70–0.99).

### Calculation of perfusion fraction in SCWEs

The two readers independently drew regions of interest (ROIs) encompassing the entire SCWE on the postcontrast T1WI. A second set of ROIs were then drawn within the tumor on the cerebral blood flow (CBF) map to mark areas showing similar to increased perfusion compared with normal-appearing cortical grey matter. This was accomplished using a matching slice in the postcontrast T1WI MR imaging, and only areas of increased perfusion within the contrast-enhancing tumor were included. The perfusion fraction was calculated by dividing the area of the high perfusion by the area of contrast enhancement.

$$\text{Perfusion fraction }\left(\%\right)=\frac{{\text{High perfusion area}}_{\text{ROI}}\text{ on CBF map}}{{\text{Enhancing area}}_{\text{ROI}}\text{ on post}-\text{contrast T}1\text{WI }}\;\times \;100$$

**Fig 2:**
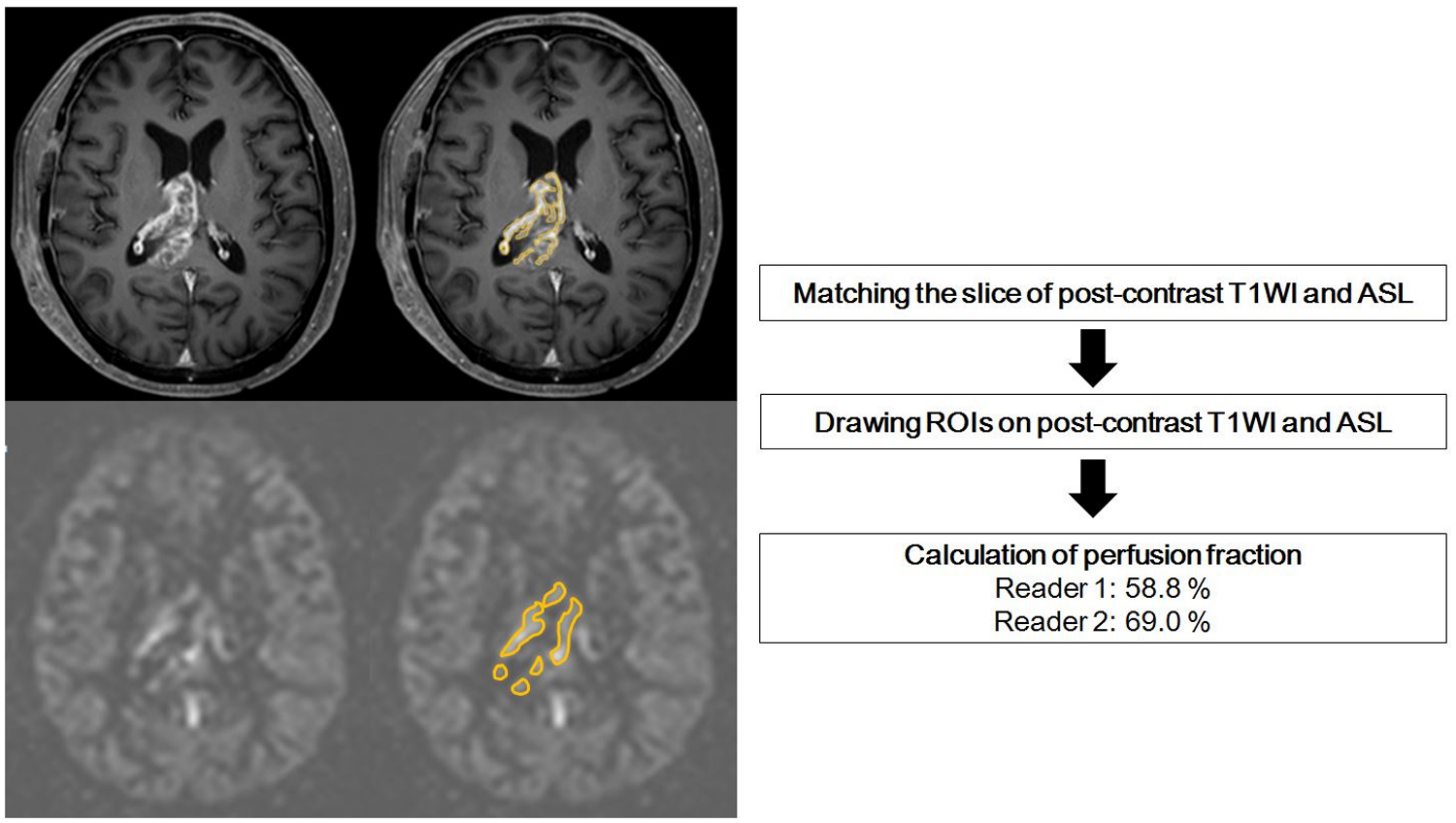
Accrual process for determining the perfusion status of surgical cavity wall enhancements. The perfusion fraction was calculated by dividing the area of the high perfusion on ASL MR imaging by the area of contrast-enhancement on postcontrast T1-weighted imaging. The perfusion fraction was 58.8% for reader 1 and 69% for reader 2.

The accrual process for volume fraction calculation is summarized in Fig 2.

### Statistical analysis

All continuous variables were initially assessed for normality using the Kolmogorov-Smirnov test. Imaging characteristics of SCWEs were compared between progression and non-progression group patients using Fisher’s exact test or the chi-square test for categorical data, with the Student’s independent *t* test used for non-categorical data.

Univariate and multivariate analyses of TTP were performed using a Cox proportional hazards model. These statistical analyses were used to evaluate the association between clinical outcomes and covariates of age, surgical extent, enhancement pattern, presence of measurable contrast enhancement, and perfusion status. Backwards elimination with a 0.10 significance level was used to develop a multivariate model. In terms of TTP related to the perfusion status of the SCWE, survival curves were created using Kaplan-Meier analysis, and a log-rank test was used to compare differences.

For the perfusion fraction of SCWEs, univariate and multivariate analyses of TTP were performed. Additionally, standard linear regression analysis was employed to evaluate the relationship between perfusion fraction and TTP.

The inter-reader agreement of the perfusion fraction was assessed using the intraclass correlation coefficient (ICC) using a two-way random effect model with consistency assumption and classified according to common criteria as excellent (ICC > 0.75), fair to good (ICC = 0.40–0.75), or poor (ICC ≤ 0.40) [20]. *P* < 0.05 was considered statistically significant. Statistical analyses were performed using MedCalc 15.6.1 (MedCalc Software, Mariakerke, Belgium).

## Results

### Clinical characteristics of the study patients

Table 1 summarizes the clinical characteristics of the study patients. The mean age of these 60 enrolled patients (33 men, 27 women) at the initial diagnosis was 58.5 ± 9.8 years. The Karnofsky Performance Scale score was dichotomized as < 70 or ≥ 70 (the ability of a person to perform usual activities) [21]. The mean number of cycles of adjuvant TMZ therapy after ASL MR imaging was 5.9. By the time of the last assessment (February 2, 2017), 42 of the 60 patients (70%) had tumor progression.

**Table 1:** Clinical characteristics and outcome of the study patients.

Characteristics	N = 60
Age (years, mean ± SD)	58.5 ± 9.8
Sex	
Male	33
Female	27
Karnofsky performance score	
<70	8
≥70	52
Surgery	
Partial resection	44
Gross total resection	16
Number of adjuvant TMZ cycles after MR imaging	5.9 ± 2.4
Dose of adjuvant TMZ (mg)	334 ± 34
Mean size of the surgical cavity (bidimensional, mm^2^)	296 ± 329
Pattern of contrast enhancing lesion	
Non-nodular	17
Nodular	43
Presence of measurable enhancing lesion	20
Clinical outcome	
Median TTP (months)*	10 (6–22)
Mean TTP (months)	16 ± 15
No progression during follow up (censored)	18

Key: SD, standard deviation; TMZ, temozolomide; TTM, time-to-progression.

*Data are median values, with interquartile range shown in parentheses. Unless otherwise indicated, data are expressed as a mean ± standard deviation.

### Image analysis of SCWEs

The imaging characteristics of the SCWEs are summarized in Table 2.

**Table 2:** Comparison of SCWE imaging characteristics.

Characteristics	Total (n = 60)	Progression group (n = 42)	Non-progression group (n = 18)	*P* value
Pattern of SCWE				0.23
Non-nodular	17	10	7	
Nodular	43	32	11	
Presence of measurable enhancing lesion	20	16	4	0.23
Perfusion status				0.25
Increased	44	29	15	
Decreased	16	13	3	
Perfusion fraction (%)				
Reader 1	38.9 ± 33	34.1 ± 32	50.1 ± 33	0.08
Reader 2	35.4 ± 35	28.9 ± 24	50.6 ± 36	**0.03**

SCWE, surgical cavity contrast enhancement.

Neither the contrast enhancement patterns, nor the presence of a measurable enhancing lesion at the SCWEs were significantly different between the two groups. On visual analysis of ASL images, increased perfusion at SCWEs was more commonly observed in the non-progression group than in the progression group, but this difference did not reach statistical significance. The perfusion fraction was higher in the non-progression group, which was significant for reader 2 (*P* = 0.03) and borderline significant for reader 1 (*P* = 0.08). The reproducibility of the perfusion fraction between the two readers was excellent (ICC = 0.76, 95% CI = 0.61–0.86).

### TTP according to the imaging characteristics of SCWEs

The median observation period was 16 months (interquartile range, 10–30 months). The median TTP was 10 months (interquartile range, 6–22 months). Analysis using the univariate Cox model revealed that positive perfusion on visual analysis was significantly associated with a longer TTP (hazard ratio [HR] = 0.49, *P* = 0.04). A perfusion fraction with a higher volume fraction was found to be associated with a longer TTP for both readers (HR = 0.98, *P* = 0.021 for reader 1; HR = 0.98, *P* = 0.005 for reader 2). The patients’ characteristics, enhancement pattern, and presence of a measurable enhancing lesion were not predictive for TTP.

The multivariate Cox model showed that a younger age (*P* = 0.03), gross total resection (*P* = 0.013), positive perfusion status (*P* = 0.02), and high perfusion fraction (*P* = 0.001 for reader 1 and < .001 for reader 2) remained an independent predictor of a longer TTP (Table 3).

**Table 3:** Cox proportional model analysis of time-to-progression.

Variable	Univariate analysis		Multivariate analysis	
	HR (95% CI)	*P* value	HR (95% CI)	*P* value
Age (years)	1.02 (0.99–1.04)	0.13	1.02 (0.99–1.05)	0.06
Male sex	0.95 (0.52–1.76)	0.88		
KPS (binary)	0.97 (0.41–2.30)	0.94		
Surgical method				
Partial resection	0.81(0.41–1.63)	0.56		
Gross total resection	0.54 (0.24–1.19)	0.12	0.72 (0.48–1.07)	0.11
Dose × number of adjuvant TMZ	1.00 (0.99–1.00)	0.96		
SCWE pattern				
Non-nodular enhancement	0.51 (0.27–1.13)	0.10		
Presence of measurable-enhancing lesion	1.47 (0.78–2.78)	0.23		
SCWE Perfusion using				
**positive** perfusion state	0.49 (0.25–0.96)	**0.04**	0.33 (0.15–0.68)	**0.02**
Perfusion fraction (reader 1)	0.98 (0.98–0.99)	**0.021**	0.97 (0.97–0.99)	**0.001**
Perfusion fraction (reader 2)	0.98 (0.98–0.99)	**0.005**	0.98 (0.97–0.99)	**< 0.001**

Note: KPS was either < 70 or ≥ 70. Key: TMZ = temozolomide; TBF = tumour blood flow.

**Fig 3:**
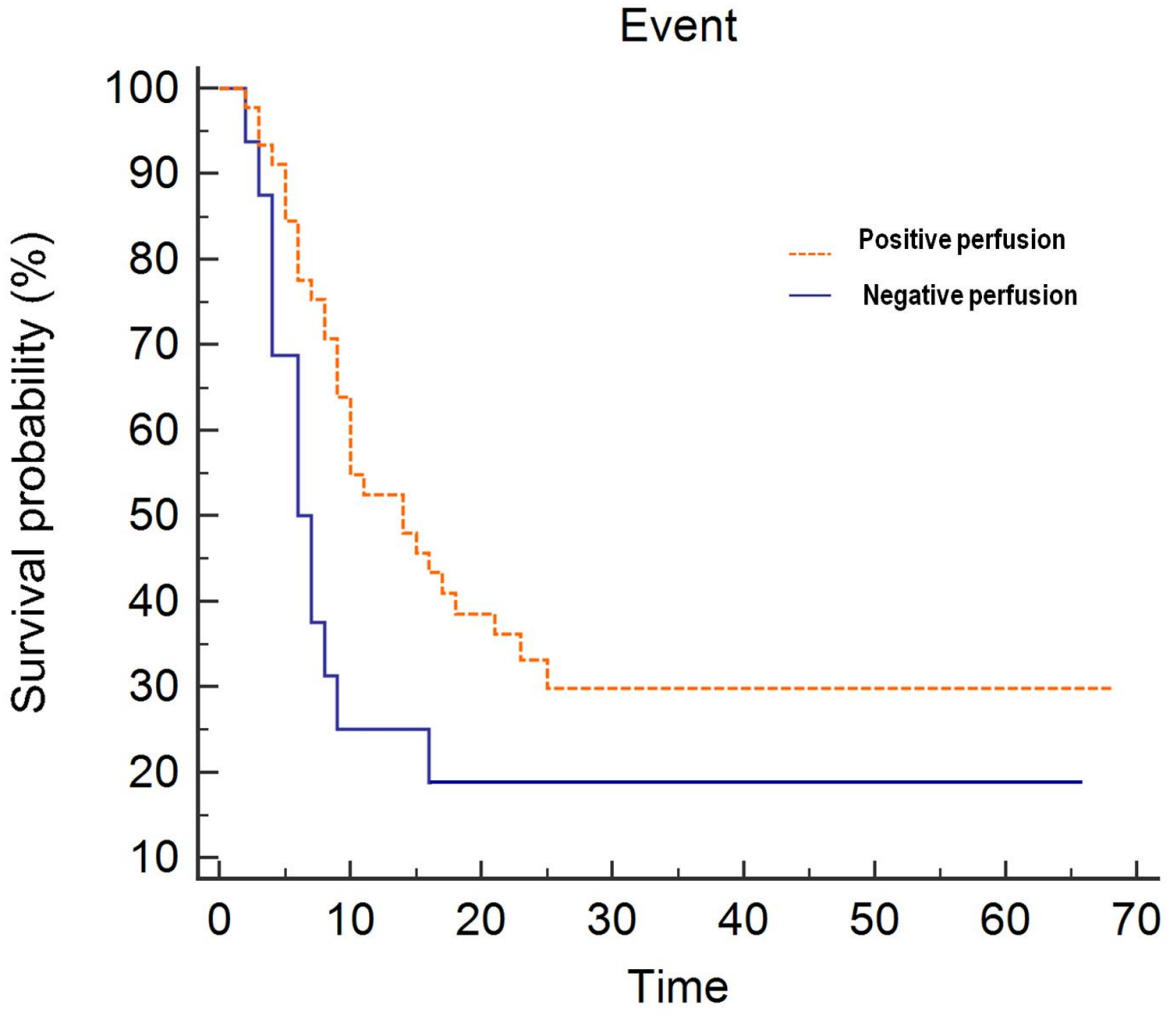
Kaplan-Meier survival curves showing clinical outcome comparisons in the increased- and decreased-perfusion groups (median time-to-progression, 13 vs. 29 months, *P* = 0.036).

**Fig 4:**
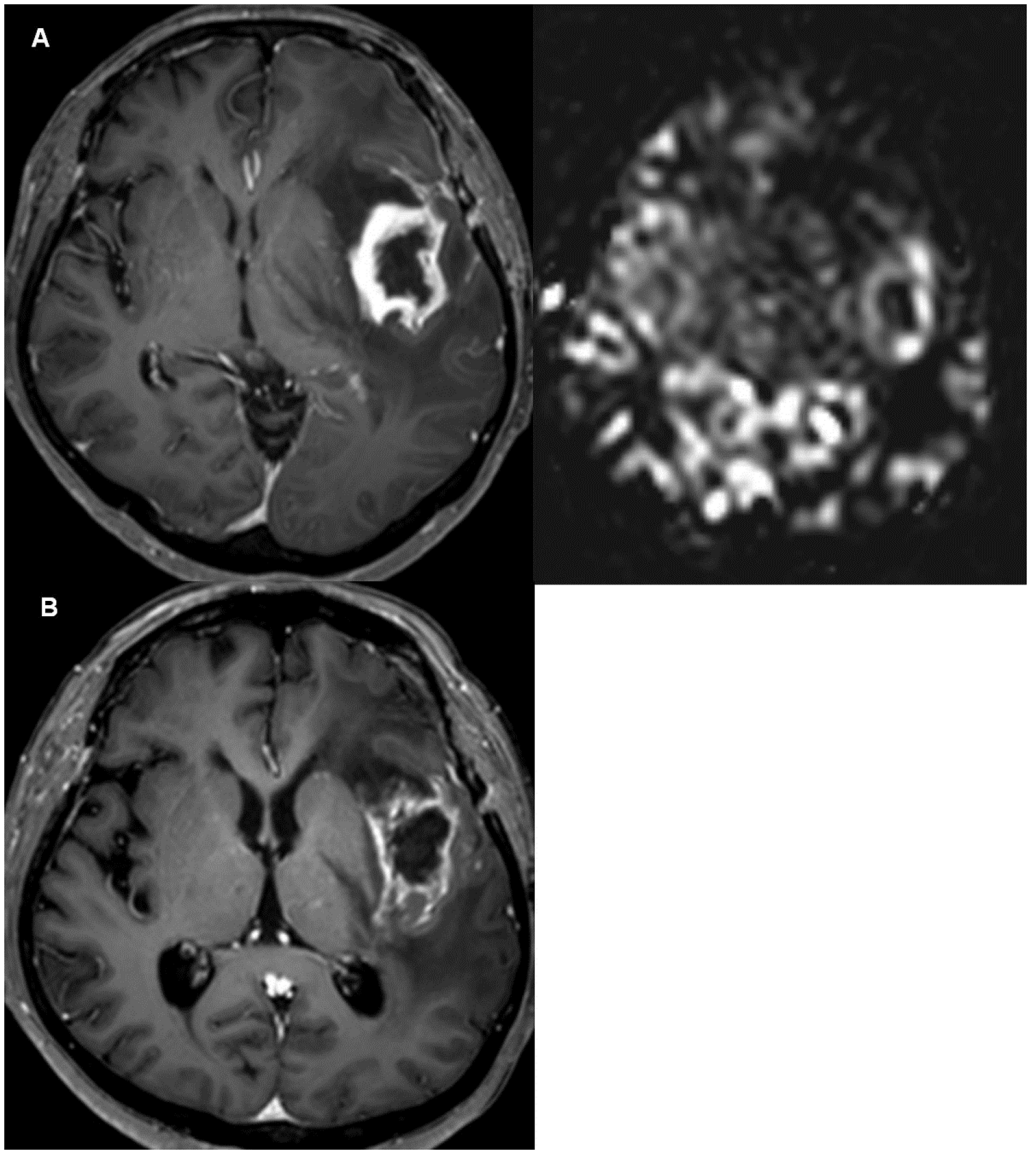
*A*, MR images from a 53-year-old male patient with glioblastoma taken 3 weeks after concurrent chemo-radiation. The contrast-enhanced MRI (left) and ASL (right) showed positive perfusion along surgical cavity wall enhancement. *B*, Follow-up images taken 4 months later after additional 5 cycles of temozolomide show that the SCWE becomes stabilized.

**Fig 5:**
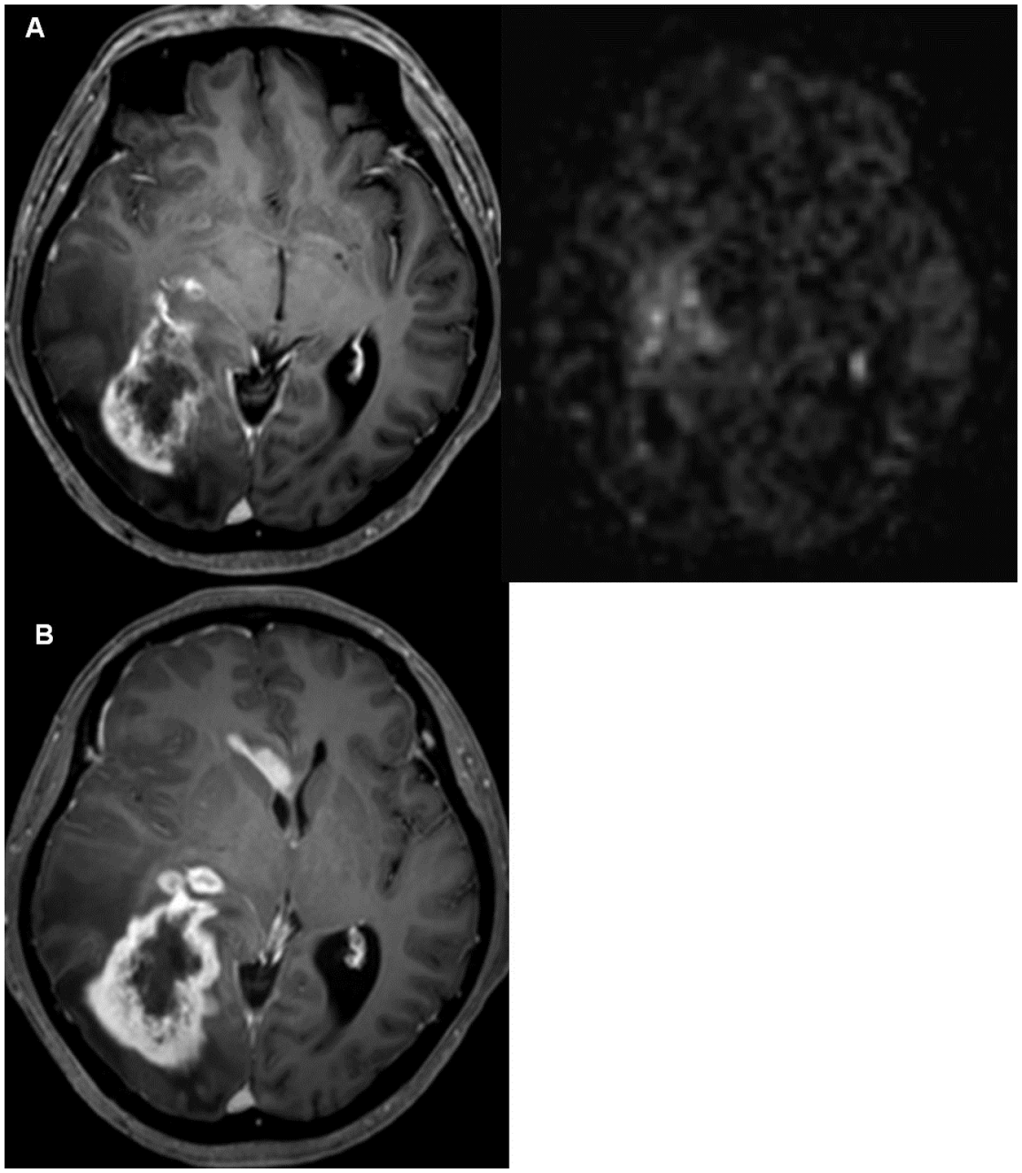
*A*, MR images from a 63-year-old female patient with glioblastoma taken 2 weeks after concurrent chemo-radiation. The contrast-enhanced MRI (left) and ASL (right) showed negative perfusion in the surgical cavity wall enhancement (SCWE). *B*, Follow-up images taken 2 months later show evident tumour progression. Note that enhancing lesions increased mostly at the regions of previously negative perfusion, the posterior portion of SCWE.

The Kaplan-Meier method with log-rank testing indicated the same trend, namely, that the increased-perfusion group was associated with a longer TTP compared with the decreased-perfusion group (median TTP = 13 vs. 29 months, indicating a maximum 2.2-fold increase in the median TTP; *P* = 0.036; Fig 3). A representative case is shown in Figs 4 and 5.

Standard linear regression coefficients were calculated to determine the association between the perfusion fraction and the TTP. Linear regression analysis revealed an *R*^2^ = 0.18 (*P* < .001; slope = 19.3; 95% CI: 8.44–30.06) for reader 1 and an *R*^2^ = 0.17 (*P* < .001; slope = 17.5; 95% CI: 7.43–27.61). The results are presented in Table 4.

**Table 4:** Results of linear regression between the perfusion fraction and TTP.

Perfusion fraction	Regression Coefficient	95% CI	Intercept	95% CI	*R*^2^
Reader 1	19.25	8.44–30.06	8.81	3.29–14.32	0.18
Reader 2	17.52	7.43–27.61	10.09	5.03–15.16	0.17

## Discussion

Our present study suggests that the perfusion status of SCWEs on post-CCRT MR imaging could be a significant predictor of the TTP in glioblastoma patients. In particular, the patients with a SCWE with positive perfusion on ASL showed a longer TTP. Semi-quantitative analysis also indicated a longer TTP in patients with a higher perfusion fraction. The enhancement pattern or presence of a measurable enhancing lesion was not found to be significant TTP predictors. Based on our results, we suggest that an increased perfusion may be associated with a longer TTP in glioblastoma and ASL MR imaging may be used as a predictive imaging biomarker for the post-CCRT status- in these patients before adjuvant TMZ therapy.

We hypothesized that ASL would become a predictive biomarker for subsequent TMZ therapy, and identify individuals who are more likely to respond to TMZ. Our patients received adjuvant TMZ after ASL MR imaging (mean number of adjuvant TMZ cycles after MR imaging = 5.9). Based on our results, increased perfusion may be associated with a longer TTP for glioblastoma. We speculate that an area with increased perfusion could possibly reflect enhanced TMZ delivery, whereas impaired perfusion would severely compromise the delivery of TMZ. It may seem paradoxical to expect a favourable outcome in the positive-perfusion group, but observations from preclinical data have indicated that vascular normalization [14, 15] of an abnormal tumor vasculature results in increased perfusion, which allows more efficient delivery of combined therapeutic agents [13, 16]. This observation is further strengthened by the findings of recent clinical trials that combination therapy achieving vascular normalization is associated with a favourable outcome in head and neck cancer [22] and in metastatic colorectal, renal, and lung cancer [23–25]. In the present study, we speculated that areas of increased perfusion compared with contralateral grey matter may reflect a region of enhanced perfusion of a viable tumor or a post-treatment tissue change. Although the mechanism underlying this is not clear, we assume that an area with increased perfusion could possibly reflect enhanced TMZ delivery, whereas low and impaired perfusion from excessive damage to the vasculature would severely compromise the delivery of chemotherapeutics.

We used semi-quantitative calculations of increased perfusion areas by comparison with normal grey matter on ASL MR imaging. More quantitative methods of analysis, such as absolute CBF calculation [26] is available, but quantitative studies have limited applicability in a clinical setting due to their low precision; previous studies have reported low intra- and inter-session reproducibility indexes for quantitative measurement as low as 18–25% [27]. Our current results demonstrate an improve robustness in the the dichotomization of increased- and decreased-perfusion groups, with an agreement of visual analysis of kappa 0.84. In addition, semiquantitative analysis of perfusion does not require for excessive post-processing and shows sufficient reproducibility (ICC = 0.76) to be adopted in clinical practice.

The enhancement pattern or presence of a measurable enhancement was not found to be a significant predictor of the TTP. Previous studies using postoperative MR imaging have reported that thick or nodular enhancement patterns had a poorer prognosis than thin enhancements, according to a thickness criterion of 5 mm [2–4]. In addition, a recent study by Kim et al. [28] of newly developed non-measurable enhancing lesions after CCRT reported a similar result, namely that the progression group showed frequent thick (≥ 3 mm) or nodular (≥ 5 mm) enhancements. Our current study differs from those of previous studies however because only thick wall enhancement (≥ 5 mm) was included in our present analysis to assess the significance of perfusion at SCWEs. In addition, unlike other reports, we analyzed a data set within a narrow time period post-CCRT but prior to adjuvant TMZ therapy.

Pseudoprogression can be a confounder in the assessment of an adjuvant TMZ treatment response. Pseudoprogression is diagnosed when a post-radiation MRI indicates an increase in contrast enhancement that subsides with time, without any change in therapy, and may therefore have represented radiation change [1, 29]. However, pseudoprogression has also shown decreased perfusion on ASL in previous studies [10, 11] and its possible confounding effect of pseudoprogression is unlikely to have caused the association between decreased perfusion and shorter TTP in our current study.

The present study had several limitations of note, beyond those associated with retrospective analyses. First, the study population was relatively small. Although we detected statistically significant results for the perfusion status of SCWEs, larger populations are needed to strengthen this statistical power, particularly with regard to the enhancement pattern. However, our patient group was unique in that we performed ASL imaging within 1 month of CCRT completion, providing a relatively homogenous group with a narrow time period for the determination of TTP. Second, we did not include the MGMT promoter methylation status in the analysis, which may synergistically improve clinical outcomes in TMZ therapy. The MGMT promoter methylation status is a determinant of chemosensitivity, whereas increased tumor perfusion is related to efficient drug delivery. Hence, the potential predictive value of combining ASL imaging and MGMT promoter methylation status should be a future study topic.

In conclusion, an assessment of perfusion in early post-treatment MR imaging can stratify the TTP in patients with glioblastoma. ASL MR imaging may be used as a predictive imaging biomarker for the post-CCRT status- in these patients before adjuvant TMZ therapy, and notably, increased perfusion in SCWEs can become a predictor of a longer TTP.
